# Comparison of PCWP and LVEDP Measurements in Patients with Severe Aortic Stenosis Undergoing TAVI—Same Same but Different?

**DOI:** 10.3390/jcm11112978

**Published:** 2022-05-25

**Authors:** Elke Boxhammer, Moritz Mirna, Laura Bäz, Brunilda Alushi, Marcus Franz, Daniel Kretzschmar, Uta C. Hoppe, Alexander Lauten, Michael Lichtenauer

**Affiliations:** 1 Department of Internal Medicine II, Division of Cardiology, Paracelsus Medical University of Salzburg, 5020 Salzburg, Austria; e.boxhammer@salk.at (E.B.); m.mirna@salk.at (M.M.); u.hoppe@salk.at (U.C.H.); 2 Universitäts-Herzzentrum Thüringen, Clinic of Internal Medicine I, Department of Cardiology, Friedrich Schiller University, 07743 Jena, Germany; laura.baez@med.uni-jena.de (L.B.); marcus.franz@med.uni-jena.de (M.F.); daniel.kretzschmar@med.uni-jena.de (D.K.); 3 Department of General and Interventional Cardiology and Rhythmology, Helios Hospital Erfurt, 99089 Erfurt, Germany; brunilda.alushi@helios-gesundheit.de (B.A.); alexander.lauten@charite.de (A.L.); 4 Deutsches Zentrum für Herz-Kreislauf-Forschung (DZHK), Standort Berlin, 10785 Berlin, Germany

**Keywords:** aortic valve stenosis, left ventricular end-diastolic pressure, pulmonary capillary wedge pressure, right heart catheter, TAVI

## Abstract

Background: Pulmonary capillary wedge pressure (PCWP) and left ventricular end-diastolic pressure (LVEDP) are often used as equivalents for determination of pulmonary hypertension (PH). PH is a comorbidity in patients with severe aortic valve stenosis (AS) and associated with limited prognosis. The aim of the study was to examine the role of differentiated classification basis of PCWP and LVEDP in patients planning for transcatheter aortic valve implantation (TAVI). Methods: 284 patients with severe AS completed a combined left (LHC) and right heart catheterization (RHC) as part of a TAVI planning procedure. Patients were categorized twice into subtypes of PH according to 2015 European Society of Cardiology (ESC) guidelines—on the one hand with PCWP and on the other hand with LVEDP as classification basis. PCWP-LVEDP relationships were figured out using Kaplan-Meier curves, linear regressions and Bland-Altman analysis. Results: Regarding 1-year mortality, Kaplan-Meier analyses showed similar curves in spite of different classification bases of PH subtypes according to PCWP or LVEDP with exception of pre-capillary PH subtype. PCWP-LVEDP association in the overall cohort was barely present (R = 0.210, R^2^ = 0.044). When focusing analysis on PH patients only a slightly increased linear regression was noted compared to the overall cohort (R = 0.220, R^2^ = 0.048). The strongest regression was observed in patients with creatinine ≥ 132 µmol/L (R = 0.357, R^2^ = 0.127) and in patients with mitral regurgitation ≥ II° (R = 0.326, R^2^ = 0.106). Conclusions: In patients with severe AS, there is a weak association between hemodynamic parameters measured by LHC and RHC. RHC measurements alone are not suitable for risk stratification with respect to one-year mortality. If analysis of hemodynamic parameters is necessary in patients with severe AS scheduled for TAVI, measurement results of LHC and RHC should be combined and LVEDP could serve as a helpful indicator for risk assessment.

## 1. Introduction

PH is a very common concomitant disease in patients with valvular diseases, especially severe AS or severe mitral valve regurgitation. However, not only valvular heart diseases, but also diseases with associated systolic or diastolic dysfunction of the left ventricle or congenital cardiomyopathies belong to World Health Organization (WHO) Group 2 of PH [1]. This group is also called “PH due to left heart diseases” and is prognostically relevant with regard to long-term survival [2].

In order to accurately diagnose PH according to current ESC guidelines [3] and to classify it into subtypes, RHC data with a determination of mPAP as well as PCWP are meaningful. An mPAP ≥ 25 mmHg is generally considered as pulmonary hypertension; further subdivision into pre- and post-capillary PH is determined by PCWP. PCWP itself is an indirectly measured, important parameter for determining left ventricular filling pressure. This can, of course, also be obtained directly by LHC via measurement of LVEDP, which is the gold standard [4]. Under physiological conditions with mitral valve opened, PCWP is equivalent to LVEDP. Therefore, one could bravely claim to use PCWP and LVEDP simultaneously for grading PH [5].

In a setting of severe AS, there is an increase in LVEDP due to chronic pressure loading of the left ventricle and associated successive dyscontraction. This change in hemodynamics leads consecutively to an increase in pressure in the left atrium and thus to an increase in PCWP [6]. Following this causal chain, one could argue that even in severe AS, PCWP could be a useful surrogate for LVEDP, thus avoiding the risky retrograde valve passage in calcified aortic valve to preserve LVEDP.

The aim of the present study was to subdivide patients with severe AS scheduled for TAVI procedure into corresponding PH subtypes with regard to PCWP and LVEDP values and to compare them with respect to 1-year mortality. Additionally, we sought to investigate the association of PCWP and LVEDP by means of linear regression and Bland-Altman analysis.

## 2. Methods

### 2.1. Patient Population

Between 2010 and 2015, 549 patients with severe, primary degenerative AS were enrolled in the current study. In all, 225 of them received left and right heart catheterization with a combined measurement of PCWP and LVEDP. Patients without RHC measurements, without LVEDP values, with moderate-to-high mitral valve stenosis and under invasive ventilation at the time of examination were excluded. The present study—approved by the local Ethic Committee of University Hospital of Jena (No.: 3237-09/11)—was carried out with principles of Declaration of Helsinki and Good Clinical Practice.

### 2.2. Procedure of RHC and LHC

From 2010 to 2015, combined performance of RHC and LHC was the standard procedure for TAVI evaluation at University Hospital of Jena.

Patients underwent RHC by using a standardized procedure via femoral vein access and six French Swan Ganz catheters (Corodyn P1, B. Braun, Melsungen, Germany). Metek Software (Metek, Elmshorn, Germany) was used for all calculations. Pressure curves were measured using fluid-filled catheters connected to pressure transducers. All pressures were calibrated using mid thoracic level and obtained, hemodynamic data were directly imported from the charts into the electronic processing system. Right atrial pressure, right ventricular pressure, systolic pulmonary artery pressure (sPAP; mmHg), mean pulmonary artery pressure (mPAP; mmHg), diastolic pulmonary artery pressure (dPAP; mmHg) and pulmonary capillary wedge pressure (PCWP; mmHg) were recorded. Calculations of pulmonary vascular resistance (PVR; Wood units (WU)) and diastolic pressure gradient (DPG; mmHg) were performed. DPG_PCWP_ was calculated as the difference between dPAP and PCWP and DPG_LVEDP_ as the difference between dPAP and LVEDP. TPG_PCWP_ was obtained as mPAP minus PCWP and TPG_LVEDP_ as mPAP minus LVEDP. Cardiac output (CO) was assessed by using the modified Fick method with estimated oxygen consumption and was indexed to the body surface area to calculate the cardiac index.

After completion of RHC measurements, LVEDP was analyzed via LHC directly by placing five or six French catheters via femoral artery access into the left ventricle after successful retrograde passage of the aortic valve. Patients for whom retrograde aortic valve passage due to extensive valve calcification was not possible were excluded from the study. In order to afford better comparability between PCWP and LVEDP, both PCWP and LVEDP were measured by taking an average of A and V wave. This measurement method was used to provide some balance between the constellations “large a wave” (patients with LV systolic dysfunction, diastolic dysfunction and volume overload) and “large v wave” (patients with mitral valve and tricuspid valve regurgitation). In patients with currently documented sinus rhythm in recorded ECG, the results of five respiratory cycles were determined. In the presence of atrial fibrillation, 10 respiratory cycles were standardized.

### 2.3. Hemodynamic Definitions of PH

Following the current valid ESC guidelines of 2015, an invasive mPAP < 25 mmHg led to exclusion of PH, whereas PH was defined when mPAP was ≥ 25 mmHg. Patients with PH were further divided into pre-capillary PH (prec-PH) by PCWP/LVEDP ≤ 15 mmHg, isolated post-capillary PH (ipc-PH) by PCWP/LVEDP > 15 mmHg, PVR ≤ 3 WU and DPG < 7 mmHg and combined post- and pre-capillary PH (cpc-PH) by PCWP/LVEDP > 15 mmHg, PVR > 3 WU and DPG ≥ 7 mmHg. In addition, a special form, called borderline post-capillary PH (borderlinepc-PH), was established to classify patients who had PCWP/LVEDP > 15 mmHg, PVR ≤ 3 WU and DPG ≥ 7 mmHg or PVR > 3 WU and DPG < 7 mmHg. This subgroup does not appear in the current ESC guidelines but attempts to fill a scientific gap, as some patients in this study with this “borderline constellation” do not meet the criteria of either ipc-PH or cpc-PH.

A new PH definition was suggested at the 6th World Symposium 2018 in Nice, which has not found its way into the ESC guidelines yet [7]. In this definition, the mPAP cut-off value was decreased from ≥25 mmHg to >20 mmHg. PCWP of <15 mmHg vs. ≥15 mmHg remains as an unchanged criterion to differentiate between pre-capillary and post-capillary PH. DPG is to be dropped as a criterion for the classification between ipc-PH and cpc-PH. Slight changes also affected the ipc-PH as well as the cpc-PH classification. The PVR criterion was changed to <3 WU instead of ≤3 WU and from >3 WU to ≥3 WU, respectively. The classification of pre-capillary PH should also be expanded to include the obligatory PVR criterion ≥3 WU in addition to PCWP ≤ 15 mmHg.

In this study, the currently still valid ESC guidelines were deliberately used, as they allow a good comparability with previously published studies, which also used the PH classification from 2015.

### 2.4. Transthoracic Echocardiography

Commercial ultrasonic devices (iE33 and Epic 5; Philips Healthcare, Hamburg, Germany) were used for performing transthoracic echocardiography. Severe AS was classified according to current guidelines of ESC measuring. The biplane Simpson’s method was applied to obtain left ventricular ejection fraction (LVEF). Spectral and color-Doppler images were used to graduate mitral, aortic, and tricuspid valve regurgitation in minimal, mild (I), moderate (II) and severe (III). Maximal tricuspid regurgitant jet velocity combined with central venous pressure using a diameter of inferior vena cava was used to calculate systolic pulmonary artery pressure (sPAP).

### 2.5. TAVI Procedure

The indication for TAVI was made by a multidisciplinary heart-team consisting of cardiologists and cardio surgeons. TAVI procedure was performed as previously described [8]. Aortic valve prosthesis of Edwards Sapien (Edwards Lifesciences, Irvine, CA, USA), CoreValve (Medtronic, Minneapolis, MI, USA,) or Jena Valve (JenaValve Technology Inc., Irvine, CA, USA) was used for transfemoral TAVI. Transapical approach was performed with Jena Valve and Edwards Sapien.

### 2.6. Clinical Follow Up and Study End Point

Follow up was performed at 12 months after TAVI by outpatient examination. One-year mortality was the primary endpoints of this study.

### 2.7. Statistical Analysis

Frequencies/percentages were used for nominal and ordinal data and mean ± standard deviation (SD) for metric variables. Baseline characteristics of different PH subtypes were examined for normal distribution by Kolmogorov-Smirnov test and were compared using analysis of Chi-Square-Test for categorical data or analysis of variance (ANOVA) for metric data. Survival curves were carried out with Kaplan-Meier methods; for comparison of different curves the log-rank test was used. Calibration of PCWP to LVEDP was assessed using linear regression and Bland-Altman analysis. A Statistical analysis was done using SPSS 25 (SPSS Inc, Chicago, IL, USA).

## 3. Results

### 3.1. Patient Population

**Figure 1:**
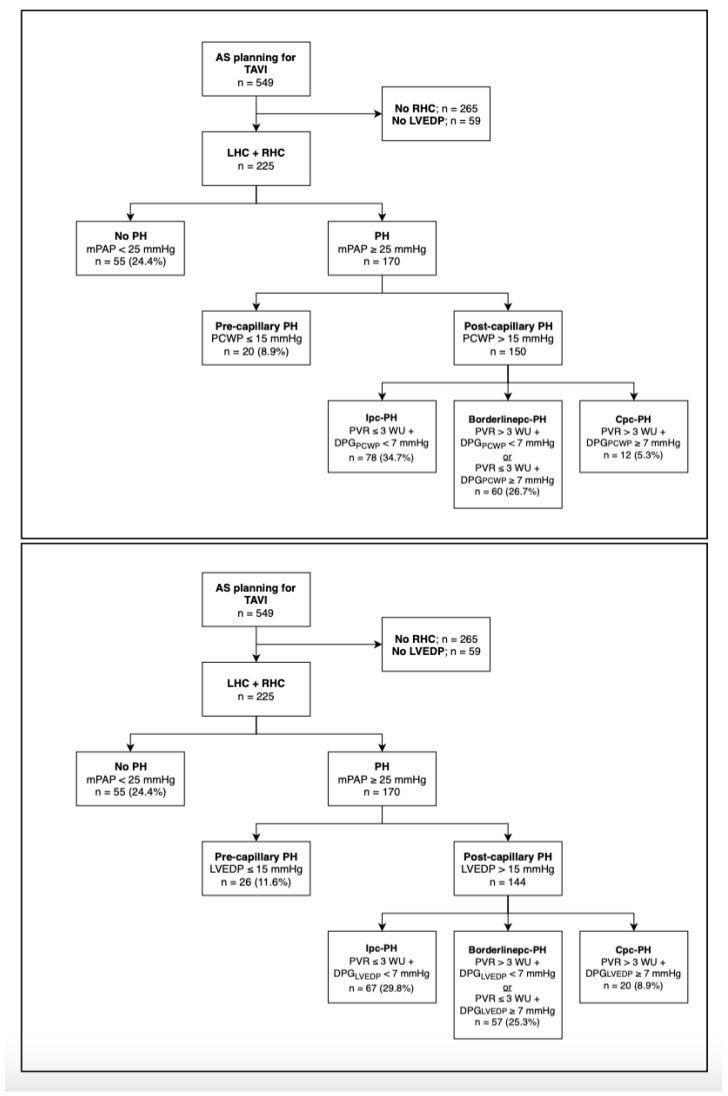
Patient disposition in study cohort. AS: aortic stenosis; TAVI: transcatheter aortic valve implantation; LHC: left heart catheterization; RHC: right heart catheterization; LVEDP: left ventricular end-diastolic pressure; PH: pulmonary hypertension; mPAP: mean pulmonary arterial pressure; PCWP: pulmonary capillary wedge pressure; Ipc-PH: isolated post-capillary pulmonary hypertension; Borderlinepc-PH: borderline post-capillary pulmonary hypertension; Cpc-PH: combined post- and pre-capillary pulmonary hypertension; PVR: pulmonary vascular resistance; DPG: right diastolic pressure gradient.

Flow charts of the study group regarding differentiated classification bases (PCWP vs. LVEDP) are carried out in Figure 1.

A total of 549 patients with severe AS were scheduled for minimally invasive aortic valve replacement via TAVI at the University Hospital of Jena from 2010 to 2015. In all, 225 of them received combined RHC and LHC with documented PCWP and LVEDP measurements. Both PCWP and LVEDP were used as the basis of classification of PH according to current ESC guidelines. The proportion of patients without PH was 24.4%. The prec-PH in LVEDP subdivision was 11.6%, 2.7% higher than in PCWP subdivision (8.9%). For the more advanced classification of post-capillary PH, borderlinepc-PH subtype was almost balanced with 26.7% (PCWP) and 25.3% (LVEDP), whereas cpc-PH subtype was more prevalent in the LVEDP group with 8.9% vs. 5.3%, whereas ipc-PH subtype was slightly weaker with 29.8% vs. 34.7%.

**Figure 2:**
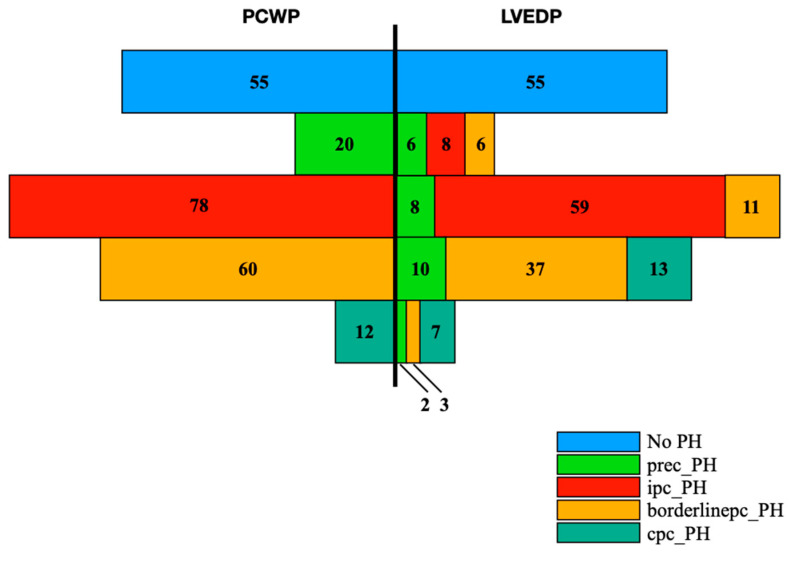
Categorization of the entire patient cohort (*n* = 225) into the respective PH subgroups according to PCWP or LVEDP. The left side shows the subgroups according to PCWP and the right side the respective re-categorization according to LVEDP. No PH: No pulmonary hypertension; Prec-PH: pre-capillary pulmonary hypertension; Ipc-PH: isolated post-capillary pulmonary hypertension; Borderlinepc-PH: borderline post-capillary pulmonary hypertension; Cpc-PH: combined post- and pre-capillary pulmonary hypertension.

To show the potential “mismatch“ of PCWP and LVEDP in terms of PH subgroup classification, Figure 2 was created.

Since the presence or absence of PH is determined purely by the mPAP, there are no relevant differences between PCWP and LVEDP in the non-PH group (blue bar) with *n* = 55 in each case. In all, 20 patients were assigned to the prec-PH group (light-green bar) after determination of PCWP. Only six (30.0%) of these 20 patients would meet the criteria of prec-PH according to LVEDP. Eight patients would have been classified in the ipc-PH group (red bar) and another six patients in the borderlinepc-PH group (orange bar). The mismatch was considerably less prominent in the ipc-PH, the borderlinepc-PH and the cpc-PH group (dark-green bar). Of the 78 patients classified into the ipc-PH group according to PCWP, 59 patients (75.6%) also fulfilled the criteria for ipc-PH according to LVEDP. Of the 60 patients assigned to borderlinepc-PH according to PCWP, 37 patients (61.7%) also showed a borderlinepc-PH subtype according to LVEDP. Among the 12 patients with classified cpc-PH after PCWP, seven patients (58.3%) also demonstrated cpc-PH after LVEDP.

### 3.2. General Characteristics and Measurements

Clinical and laboratory data, percentual distributions of concomitant diseases, echocardiographic measurements, measurements of RHC/LHC and procedural TAVI data with corresponding *p*-values are shown in Table 1.

In this context, a detailed comparison of corresponding subtypes with additional subdivision according to PCWP or LVEDP was carried out. Because there was conceivable overlap (compare Figure 2) between PCWP and LVEDP classified subgroups (for example, one patient could be assigned to both the PCWP-prec-PH and LVEDP-prec-PH subtype) comparison of means between these subtypes with respect to significance level was prohibited. Therefore, a descriptive analysis was performed in the following:

Considering the collected data, no major differences could be found within the different subgroups. However, the prec-PH subtype was an exception. Here, there was not only a clear difference in echocardiographically collected sPAP values, but also in several RHC data (sPAP, mPAP, dPAP, etc.).

Special attention should be paid to the divergence between PCWP and LVEDP within the subtypes. In the PCWP-based prec-PH subtype, PCWP value was recorded as 13.05 mmHg, whereas LVEDP value averaged with 19.75 mmHg in post-capillary PH ranges. The exact opposite constellation was shown by prec-PH subtype on LVEDP basis, where LVEDP was recorded at 11.85 mmHg, but PCWP was measured at 20.42 mmHg and thus in post-capillary PH ranges.

When focusing on PCWP and LVEDP only, 16/225 patients (7.1%) showed a PCWP ≤ 15 mmHg and an LVEDP ≤ 15 mmHg, 54/225 (24.0%) had a PCWP ≤ 15 mmHg and an LVEDP > 15 mmHg, 22/225 (9.8%) demonstrated a PCWP > 15 mmHg and an LVEDP ≤ 15 mmHg, and 133/225 (59.1%) presented a PCWP > 15 mmHg and an LVEDP > 15 mmHg.

### 3.3. Linear Regression and Bland-Altman Analysis

**Figure 3:**
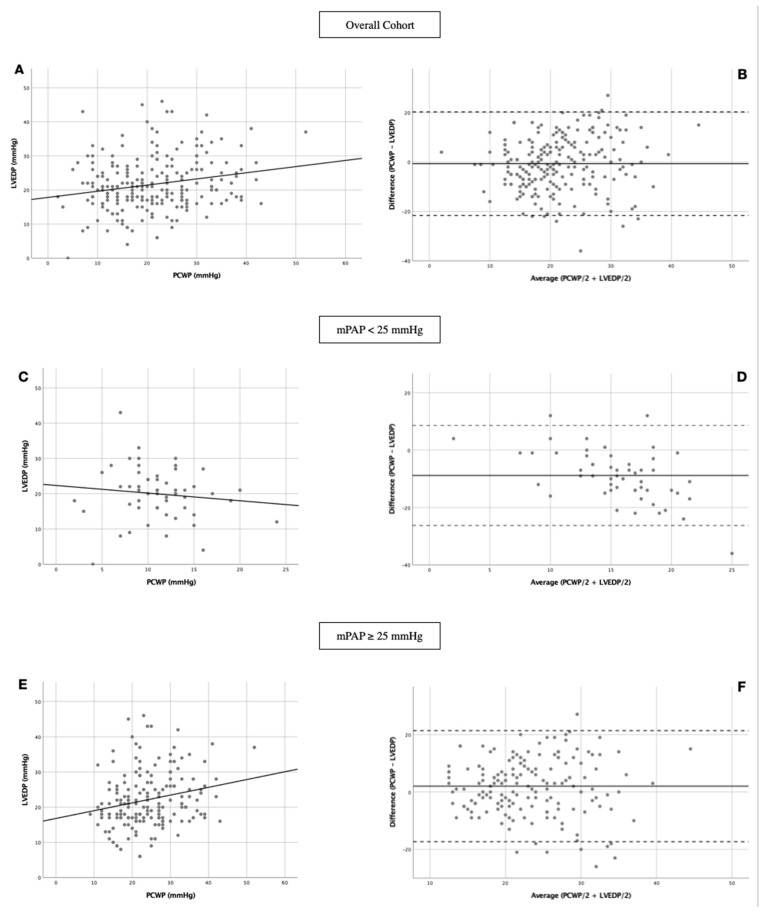
Linear regression and Bland-Altman analysis between PCWP and LVEDP. (**A**) Linear regression in overall cohort; (**B**) Bland-Altman analysis in overall cohort; (**C**) Linear regression in No PH patients; (**D**) Bland-Altman analysis in No PH patients; (**E**) Linear regression in PH patients; (**F**) Bland-Altman analysis in PH patients. LVEDP: left ventricular end-diastolic pressure; PCWP: pulmonary capillary wedge pressure; mPAP: mean pulmonary arterial pressure.

Regression analysis and Bland-Altman plots to compare relationships between PCWP and LVEDP measurements in dependence of clinically important patient characteristics are represented in Figure 3 and Table 2.

Based on the assumption that PCWP is commonly used as a surrogate for LVEDP and that both values provide information about left atrial pressure, causality was suspected, and therefore linear regression was used.

PCWP and LVEDP regression in overall cohort was weak (R = 0.210, R^2^ = 0.044, *p* = 0.002; Figure 3A). In Bland-Altmann analysis mean bias was −0.66 mmHg with 95% limits of agreement ranging from −21.65 to 20.32 mmHg (Figure 3B). This implies that PCWP underestimated LVEDP by 0.66 mmHg on average. Even after excluding 5% of the patient with the greatest difference between PCWP and LVEDP, PCWP underestimated LVEDP by 21.65 mmHg and overestimated it by 20.32 mmHg.

By restricting linear regression and Bland-Altmann analysis on No PH patients (*n* = 55) no significant relationship between PCWP and LVEDP with R = 0.117, R^2^ = 0.014 and *p* = 0.394 (Figure 3C) could be observed. PCWP underestimated LVEDP by 8.84 mmHg; limits of agreement ranged from −26.26 to 21.38 mmHg (Figure 3D).

When focusing analysis on PH patients only (*n* = 170) a slightly increased linear regression was noted compared to the overall cohort (R = 0.220, R^2^ = 0.048, *p* = 0.004; Figure 3E). In contrast to the entire cohort or No PH patients, PCWP overestimated LVEDP in PH patients by 2.02 mmHg with limits of agreement ranging from −17.34 to 21.38 mmHg (Figure 3F).

Linear regression between PCWP and LVEDP was also investigated regarding concomitant diseases (Table 2). The strongest regression was observed in patients with creatinine ≥ 132 µmol/l (R = 0.357, R^2^ = 0.127, *p* = 0.011) and in patients with mitral regurgitation ≥ II° (R = 0.326, R^2^ = 0.106, *p* = 0.001).

### 3.4. Kaplan-Meier Curves

**Figure 4:**
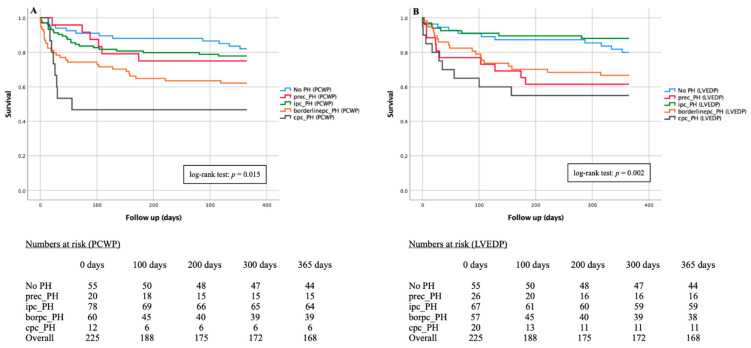
Kaplan-Meier curves of 1-year mortality with associated numbers at risk in patients with severe AS classified in PCWP- and LVEDP-PH-subtypes. (**A**) Comparison of No PH vs. PCWP-PH-subtypes; (**B**) Comparison of No PH vs. LVEDP-PH-subtypes. Prec-PH: pre-capillary pulmonary hypertension; Ipc-PH: isolated post-capillary pulmonary hypertension; Borderlinepc-PH: borderline post-capillary pulmonary hypertension; Cpc-PH: combined post- and pre-capillary pulmonary hypertension.

**Figure 5:**
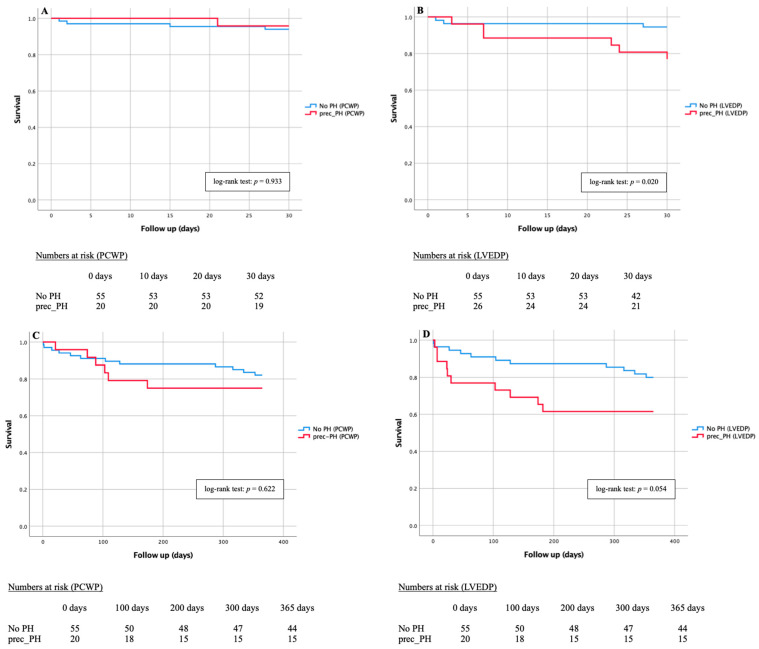
Kaplan-Meier curves of 30-day and 1-year mortality with associated numbers at risk in patients with severe AS diagnosed with pre-capillary PH via PCWP and LVEDP. (**A**) 30-day mortality of No PH vs. pre-capillary PCWP-PH; (**B**) 30-day mortality of No PH vs. pre-capillary LVEDP-PH; (**C**) 1-year mortality of No PH vs. pre-capillary PCWP-PH; (**D**) 1-year mortality of No PH vs. pre-capillary LVEDP-PH. Prec-PH: pre-capillary pulmonary hypertension.

Kaplan-Meier curves of PCWP-PH-subtypes and LVED-PH-subtypes as well as different LVEDP and PCWP values regarding 1-year-mortality are demonstrated in Figure 4. In addition, 30-day and 365-day mortality of pre-capillary PH subtype was examined separately according to PCWP and LVEDP classification (Figure 5).

During an observation period of 12 months, the primary endpoint death was reached in the PCWP-cohort (*n* = 225) a total of 57 times (25.3%)—11/55 patients without hemodynamic evidence of PH (20.0%) and 46/170 patients with PH (27.1%). 5/20 patients (25.0%) died with documented prec-PH, 14/78 (17.9%) with ipc-PH, 21/60 (35.0%) with borderlinepc-PH and 6/12 (50.0%) with cpc-PH. The Kaplan-Meier curve with division into various PH subtypes (Figure 4A) showed a significant difference in the log-rank test with a p = 0.015.

A similar picture emerged in the LVEDP-cohort (*n* = 225). Of 46 deceased PH-patients, 10/26 (38.5%) demonstrated a prec-PH, 8/67 (11.9%) an ipc-PH, 19/57 (33.3%) a borderlinepc-PH and 9/20 (25.0%) a cpc-PH. Again, the log-rank test showed statistical significance with a p = 0.002 (Figure 4B).

Due to apparent divergence of PCWP- and LVEDP-prec-PH subtypes, further Kaplan-Meier curves were added. After 30 days (Figure 5A,B) 95.0% of PCWP-prec-PH patients (19/20) and only 76.9% of LVEDP-prec-PH patients (20/26) were still alive, whereas after 365 days (Figure 5C,D), 75% of PCWP (5/20) and 61.5% of LVEDP-prec-PH patients (16/26) were still alive. In respective comparison to No PH patients, the log-rank test showed no statistical significance, especially in PCWP classification; in the LVEDP classification, *p* = 0.020 was recorded in the 30-day course and *p* = 0.054 in the 365-day course.

## 4. Discussion

The study represents one of the first to investigate the relationship between PCWP and LVED for assessment of PH in patients with severe AS and planned minimally invasive valve replacement (TAVI).

### 4.1. Prevalances of PH and PH Subtypes Concerning PCWP and LVEDP Classification

PH prevalence in patients with severe AS and available RHC and LHC data ranged from 48% to 75% [9,10,11,12,13]. In the present study, 170/225 representing 75.6% of all patients demonstrated a PH. Accordingly, these data are slightly higher than the values reported in other comparative studies. The highest PH population was shown to be 75% in the work of O’Sullivan and coauthors [9], who used TAVI data from 2007 to 2012. The current study also draws on a patient collective dating from 2010 to 2015, because during this period of TAVI history, combined RHC and LHC was still much more common than at present time. In collective of Weber et al. [10], 48% PH patients included subjects who underwent surgical valve replacement and therefore, by definition, have lower overall morbidity and thus lower PH prevalence. Nevertheless, it can be assumed that percentage distribution of PH patients presented in here is overestimated, because specification of patients, especially with available RHC data, results in a form of selection for patients with PH.

Considering subtype analysis, the studies mentioned before showed a variation between 6.2% and 16.4% for pre-capillary subtype. With 8.9% pre-capillary PH patients according to the PCWP classification (20/225) and 11.6% in the LVEDP classification (26/225), stable average values are achieved in present study. Post-capillary subtypes cannot be assessed satisfactorily because of differentiated classification bases. Schewel et al. [11] and O’Sullivan et al. focused on the pure DPG and Weber et al. and Sultan et al. [12] on the pure PVR for subdivision between ipc- and cpc-PH. Particularly, with so called borderlinepc-PH a special form of subtype was introduced in the present study. This separate subtype has the advantage of including both PVR and DPG in the classification and at the same time balancing the existing discrepancy in the ESC guidelines regarding the non-classifiability of patients with a PVR > 3 WU and a DPG < 7 mmHg or a PVR ≤ 3 WU and a DPG ≥ 7 mmHg.

### 4.2. Prec-PH via PCWP vs. LVEDP—Subgroup with Greatest Divergence in Terms of Hemodynamic Parameters and Mortality

Kaplan-Meier curves showed the strongest deviation between PCWP and LVEDP defined subtypes in the group of prec-PH patients, which is why a detailed survival analysis was further calculated. Patients with prec-PH classified according to LVEDP died not only significantly earlier, but also at a higher rate compared with those classified according to PCWP. Likewise, subjects of the LVEDP-prec-PH subtype showed significantly higher mortalities in contrast to subjects without PH, which could not be observed in the PCWP-prec-PH subtype.

This discrepancy was also found in previous PH-TAVI studies. In their prec-PH cohort, which was classified according to PCWP, Weber et al. demonstrated no significant differences in 1-year mortality between patients with prec-PH and without PH. In the study of O’Sullivan and a coworker, who used LVEDP as the basis for classification, Kaplan-Meier curves were similar to those in present study, with significantly higher mortality in the prec-PH group.

Hemodynamically, in particular with regard to RHC data, distinct differences between differently classified pre-capillary subtypes were also manifested. Thus, prec-PH patients classified according to LVEDP consistently showed higher values with respect to sPAP, mPAP, and dPAP. However, significant attention should be paid to DPG, which has already been investigated in several studies [14,15,16,17] as a predictor of survival prognosis. For example, Mazimba et al. [18] described that an increasing DPG is associated with increased mortality.

PCWP-prec-PH group showed an associated DPG_PCWP_ of 3.8 ± 2.8 mmHg. This was consistent with the study by Weber et al. in which prec-PH patients had a mean DPG of 0-5 mmHg and were also nearly identical with respect to associated Kaplan-Meier curves. However, these DPG values present themselves comparatively low when considering the study by Schewel et al. with a mean DPG value of 19.5 ± 5.9 mmHg; their prec-PH patients showed even worse survival in the Kaplan-Meier curves than the cpc-PH patients.

As the only one of the studies mentioned above, O’Sullivan et al. used LVEDP as a classification basis for PH grading and showed a DPG of 8.4 ± 6.9 mmHg in his pre-capillary cohort. In the LVEDP-prec-PH group of the present study, an associated DPG_LVEDP_ of 9.5 ± 7.3 mmHg manifested itself, indicating a good consensus not only in terms of DPG values but also considering Kaplan-Meier curves. This could be confirmed in a performed AUROC analysis (data not shown), in which an DPG_LVEDP_ ≥ 4.50 mmHg was shown to be a good discriminator of prec-PH (AUC 0.852; 95%CI 0.796-0.909; *p* < 0.001; sensitivity 0.85, specificity 0.78, Youden index 0.63).

Considering the current data, prec-PH is associated with increased mortality [19,20]. In contrast to PCWP classification, the LVEDP classification presented here not only reflects mortality of prec-PH much better, but also shows conclusive values of DPG in concordance with current literature. Therefore, one could postulate to use LVEDP instead of PCWP as a decision basis for PH determination in the context of severe AS or to extend PCWP by an additional criterion of DPG ≥ 7 mmHg.

### 4.3. Lack of Agreement between PCWP and LVEDP—Possible Reasons

However, the main crux of the present study should address the question of why there is an almost complete lack of agreement between PCWP and LVEDP. Regardless of whether the entire cohort, non-PH patients or PH patients were examined, no significant association emerged in regression analyses, and considerable scatter occurred in Bland-Altmann analyses as well. Bitar et al. [21], Hemnes et al. [22], and Halpern et al. [23] also found almost consistently little agreement between PCWP and LVEDP in different collectives. Hemnes et al. showed an exception in their work: In patients with mitral valve stenosis, a good correlation with an r = 0.91 was described between PCWP and LVEDP. Regarding mitral valve stenosis, no conclusion could be drawn in this study, because patients with corresponding mitral valve stenosis were excluded in advance.

As one of a few study groups, de Oliveira et al. [24] demonstrated in their study population of patients with pulmonary arterial hypertension a satisfying, positive correlation of r = 0.74 with respect to the ratio of PCWP and LVEDP. However, these study results can only be compared to a limited extent with the results presented here, because 79% of the patients in de Oliveira et al. had prec_PH and thus a narrowly defined collective with mainly low PCWP values (mean 12.7 ± 5.1 mmHg). Patients with severe AS as in the present patient collective suffer in a higher percentage from post-capillary PH, which is why PCWP values were significantly higher with a mean of 21.12 ± 9.16 mmHg. The larger scatter of measurement results (2–52 mmHg (present study) vs. 5–35 mmHg (de Oliveira)) can be most likely responsible for these nevertheless considerable differences regarding the PCWP-LVEDP relationship.

In order to clear possible causes for the lack of agreement between PCWP and LVEDP, it is essential to understand the hemodynamics as well as the measurement development of LVEDP and PCWP. LVEDP is generated by left heart catheterization via retrograde aortic valve passage, where it is known that pressure equalization between atrium and ventricle is generated during diastole and thus left atrial pressure can be inferred. Adequate PCWP measurement by right heart catheterization is achieved by inserting a balloon into the peripheral pulmonary arterial circulation and subsequently inflating it, creating a “standing column of blood” from the pulmonary artery through the capillary flow territory and pulmonary vein to the left atrium. Hereby, a statement about the left atrial pressure can be made as well. This roughly explains why PCWP is used as a surrogate parameter of LVEDP in clinical practice.

However, the balance of this simplified assumption can be upset by many different influencing variables. For example, mitral valve stenosis [25] and mechanical ventilation with high alveolar pressures [26] lead to a PCWP > LVEDP. Therefore, in advance both constellations were considered exclusion criteria in this study. The exact opposite constellation with PCWP < LVEDP is observed in patients with moderate to severe mitral regurgitation. Since almost half of all patients with severe AS had mitral regurgitation ≥ II° in addition to severe AS, this influencing factor for an imbalance between PCWP and LVEDP cannot be dismissed.

COPD, as present in 22.5% of the overall cohort, also leads to an increased pressure in the pulmonary circulation due to vascular remodeling [27] and thus consecutively to a PCWP > LVEDP. This also explains the near absence of linear regression between PCWP and LVEDP in COPD patients with an R = 0.088, an R^2^ = 0.008 and a *p* = 0.542.

Patients with obesity also showed higher PCWP values compared to LVEDP, most likely due to increased work of breathing [28] or chronic inflammatory responses [29]. Thus, in both Bitar et al. and the present study, regressions of PCWP and LVEDP were very weak at BMI > 30 mg/kg^2^ (R = 0.104, R^2^ = 0.011, *p* = 0.433).

Further discrepancies and thus an imbalance in PCWP-LVEDP relationship occur in patients with myocardial infarctions [30]. The extent to which myocardial infarction increases or decreases LVEDP is currently under investigation [31]. In any case, subjects with a history of myocardial infarction show a more pronounced difference between PCWP and LVEDP and thus a lower linear regression compared with non-myocardial infarction patients (R = 0.095, R^2^ = 0.009, *p* = 0.622).

Particular attention should also be paid to the PCWP-LVEDP relationship, taking into account the presence or absence of atrial fibrillation. It has already been shown in a previous study [32] that patients with atrial fibrillation had higher PCWP than LVEDP values because of the absence of left atrial contraction. Maeder et al. [33] showed in their collective of patients with severe AS and atrial fibrillation a small correlation between PCWP and LVEDP with r = 0.29. This is comparable to our weak regression analysis with an R = 0.216, an R^2^ = 0.047 and a *p* = 0.061.

An equally noteworthy observation as in Bitar et al. was observed in the present study, namely that the PCWP-LVEDP association was not only weaker in patients without PH (R = 0.117, R^2^ = 0.014, *p* = 0.394) but also not significant compared to PH patients (R = 0.220, R^2^ = 0.048, *p* = 0.004). Notably, in patients with severe AS without PH, PCWP significantly underestimated LVEDP by 8.84 mmHg, which could be explained by the fact that, in isolation, there is a ratio of PCWP < LVEDP in high-grade aortic valve stenosis. This can be explained hemodynamically by the fact that in severe AS, the consecutive hypertrophied left ventricle must apply higher filling pressures to eject stroke volume against the stenosis. Therefore, after closure of the mitral valve, filling pressures increase even further, so that left atrial pressure does not equal left ventricular pressure and thus PCWP does not equal LVEDP.

In conclusion, the PCWP-LVEDP relationship is dependent on multiple factors that would need to be included in PH classification basis to avoid misclassification. According to this study, to be able to make optimal statements and risk assessments for patients with severe AS and potential PH, it is prohibited to use PCWP as a surrogate parameter for LVEDP.

From the results of this study it can be concluded that in the best case both values should be determined by RHC and LHC in order to be able to make adequate statements about a potential PH. Before classifying a patient according to PCWP or LVEDP into the corresponding PH subgroup, it is essential to consider the patient’s hemodynamics and potential concomitant diseases (additional valvular heart diseases, lung diseases such as COPD, underlying rheumatologic diseases such as lupus erythematosus or scleroderma). Depending on the corresponding patient constellation, it is therefore left to the individual discretion to resort to the PCWP or the LVEDP as a decision criterion.

## 5. Conclusions

Direct comparison of PCWP and LVEDP in TAVI patients to assess PH was investigated for the first time in this constellation. As in other PH studies, it was shown that considering PCWP as a surrogate parameter for LVEDP should be done with some caution, even more in patients with AS, because many influencing factors could upset the physiological balance between PCWP and LVEDP.

## 6. Limitation

The present study relied on single-center data and may not reflect general practice in other centers. Additionally, technical pitfalls in RHC measurements should always be conceded in the context of hemodynamic measurements, even if examinations were performed by experienced clinical investigators. The volume status of patients also depends significantly on hemodynamic parameters. Thus, major deviations may occur in the case of an overload (incorrectly high pressure measurements) but also in the case of a diuretic overdose (incorrectly low pressure measurements). Last but not least, the figures refer to the years 2010 to 2015 and thus represent an earlier TAVI generation compared to the current patient population we see in the present clinical setting. However, this is due to the fact that RHC is no longer a standard procedure at major cardiology centers and therefore older data are used for this study.

## Figures and Tables

**Table 1:** Clinical and laboratory data, echocardiographic measurements, invasive hemodynamic profile and procedural data of study cohort by PH subtypes. BMI: body mass index; EF: ejection fraction; LVEDD: left ventricular end-diastolic diameter; LVESD: left ventricular end systolic diameter; sPAP: systolic pulmonary arterial pressure; AVA: aortic valve area; AV max: maximal velocity over aortic valve; AV dpmax: maximal pressure gradient over aortic valve; AV dpmean: mean pressure gradient over aortic valve; mPAP: mean pulmonary arterial pressure; dPAP: diastolic pulmonary arterial pressure; PCWP: pulmonary capillary wedge pressure; DPG: diastolic pressure gradient; TPG: transpulmonary pressure gradient; CO: cardiac output. LVEDP: left ventricular end-diastolic pressure.

	Overall Cohort		No PH		Prec-PH				Ipc-PH				Borderlinepc-PH				Cpc-PH				ANOVA/Chi-Square	
					PCWP		LVEDP		PCWP		LVEDP		PCWP		LVEDP		PCWP		LVEDP		PCWP	LVEDP
	*n* = 225		*n* = 55		*n* = 20		*n* = 26		*n* = 78		*n* = 67		*n* = 60		*n* = 57		*n* = 12		*n* = 20			
	Mean	SD (±)	Mean	SD (±)	Mean	SD (±)	Mean	SD (±)	Mean	SD (±)	Mean	SD (±)	Mean	SD (±)	Mean	SD (±)	Mean	SD (±)	Mean	SD (±)	*p*-Value	*p*-Value
**Clinical data**																						
Age (years)	80.89	7.03	80.53	6.52	83.90	6.02	83.77	7.30	80.36	7.56	79.67	7.13	80.70	7.00	82.44	6.20	81.83	7.21	77.80	8.24	0.328	0.010
Weight (kg)	73.96	12.70	72.05	13.53	76.50	17.44	70.62	12.28	77.58	10.74	78.60	11.62	71.05	11.77	73.19	12.63	69.42	11.18	70.15	11.24	0.010	0.007
Height (cm)	164.42	8.55	162.65	8.37	163.55	8.64	162.81	6.88	166.42	8.08	167.27	8.31	163.37	8.62	162.82	7.75	166.25	10.29	166.40	11.44	0.080	0.007
BMI (kg/m^2^)	27.35	4.29	27.19	4.40	28.54	5.94	26.69	4.74	28.05	3.79	28.14	4.07	26.64	4.20	25.59	4.40	25.08	3.20	25.32	3.28	0.067	0.107
STS-Score	4.01	2.48	3.54	2.20	4.86	3.25	4.75	3.16	3.88	2.56	3.69	2.24	4.31	2.18	4.38	2.39	4.09	2.99	4.35	3.01	0.242	0.131
EuroScore	25.53	15.74	25.11	16.91	38.53	21.34	27.21	14.57	22.94	13.19	23.35	14.21	25.68	14.58	27.87	17.36	21.43	11.13	24.73	14.29	0.002	0.605
**Laboratory data**																						
Creatinine (µmol/L)	119.00	76.60	106.45	58.93	118.47	54.30	110.12	44.45	123.41	75.59	125.29	77.54	125.19	98.79	118.19	64.25	115.83	57.84	143.75	144.91	0.750	0.388
C-reactive protein (mg/L)	19.10	34.38	12.37	24.13	16.14	20.05	30.80	39.07	20.19	37.40	19.75	36.53	25.92	43.36	18.48	30.08	15.42	20.52	20.32	49.67	0.384	0.310
Hemoglobin (mmol/L)	7.54	1.07	7.55	0.99	7.68	0.80	7.46	1.16	7.39	1.00	7.34	1.05	7.64	1.25	7.78	1.02	7.69	1.39	7.59	1.32	0.704	0.347
**Concomitant diseases**																						
Diabetes mellitus—no. (%)	134 (59.60)		31 (56.40)		11 (55.00)		15 (57.70)		42 (53.80)		39 (58.20)		40 (66.66)		33 (57.90)		10 (83.33)		16 (80.00)		0.237	0.426
Arterial hypertension (%)	205 (91.10)		51 (92.70)		19 (95.00)		25 (96.20)		69 (88.50)		59 (88.10)		55 (91.66)		52 (91.20)		11 (91.66)		18 (90.00)		0.869	0.773
Atrial fibrillation—no. (%)	76 (33.80)		18 (32.70)		9 (45.00)		8 (30.80)		26 (33.33)		25 (37.30)		18 (30.00)		18 (31.60)		5 (41.70)		7 (35.00)		0.758	0.958
Coronary heart disease ≥ 2 vessels—no. (%)	58 (25.80)		12 (21.80)		5 (25.00)		5 (19.20)		21 (26.90)		20 (29.90)		18 (30.00)		14 (24.60)		2 (16.66)		7 (35.00)		0.811	0.636
COPD—no. (%)	50 (22.20)		10 (18.20)		5 (25.00)		8 (30.80)		18 (23.10)		14 (20.90)		13 (21.66)		11 (19.30)		4 (33.33)		7 (35.00)		0.825	0.426
Myocardial infarction—no. (%)	29 (12.90)		9 (16.40)		1 (5.00)		3 (11.50)		8 (10.30)		4 (6.00)		9 (15.00)		9 (15.80)		2 (16.66)		4 (20.00)		0.643	0.321
Stroke—no. (%)	32 (14.20)		6 (10.90)		5 (25.00)		9 (34.60)		14 (17.90)		10 (14.90)		6 (10.00)		6 (10.50)		1 (8.33)		1 (5.00)		0.342	0.022
NYHA ≥ III—no. (%)	186 (82.70)		41 (74.50)		16 (80.00)		23 (88.50)		66 (84.60)		57 (85.10)		54 (90.00)		47 (82.50)		9 (75.00)		18 (90.00)		0.183	0.238
**Echocardiographic data**																						
EF (%)	57.57	16.14	61.02	15.64	61.70	10.46	56.87	13.90	57.96	15.61	57.96	15.43	52.89	17.69	58.50	16.13	55.59	18.17	44.97	18.22	0.064	0.006
LVEDD (mm)	48.70	7.81	47.38	8.17	47.26	6.84	47.72	7.04	49.20	7.01	49.97	7.43	49.43	8.05	47.74	7.50	50.58	10.74	52.16	8.85	0.465	0.092
LVESD (mm)	32.14	9.27	30.00	9.51	30.69	7.47	31.69	8.99	32.04	8.34	33.09	8.91	34.03	9.87	30.50	8.18	36.38	12.34	40.46	9.32	0.224	0.005
sPAP (mmHg)	41.55	13.79	32.61	7.66	33.15	12.35	40.29	11.81	43.91	11.64	41.40	12.47	47.25	15.55	47.47	14.86	52.40	13.85	50.60	15.98	< 0.001	< 0.001
AVA (cm^2^)	0.65	0.18	0.67	0.21	0.64	0.19	0.65	0.18	0.67	0.17	0.68	0.18	0.61	0.15	0.61	0.15	0.66	0.22	0.60	0.14	0.351	0.295
AV max (m/s)	4.34	0.71	4.51	0.69	4.37	0.46	4.20	0.63	4.30	0.63	4.33	0.65	4.23	0.86	4.35	0.76	4.33	0.73	4.09	0.82	0.281	0.153
AV dpmax (mmHg)	78.96	26.42	83.52	24.59	77.69	15.71	70.16	18.14	77.12	23.04	76.91	23.00	76.87	33.63	83.44	32.01	81.08	30.09	71.96	30.86	0.654	0.101
AV dpmean (mmHg)	47.97	16.65	51.03	16.90	47.39	11.87	42.65	18.14	46.35	13.97	47.53	14.46	47.12	20.40	49.37	19.50	48.80	18.87	43.85	18.01	0.603	0.195
Mitral regurgitation ≥ II°—no. (%)	99 (44.00)		20 (36.40)		2 (10.00)		11 (42.30)		35 (44.90)		26 (38.80)		39 (65.00)		32 (56.10)		3 (25.00)		10 (50.00)		< 0.001	0.275
Tricuspid regurgitation ≥ II°—no. (%)	95 (42.20)		19 (34.50)		6 (30.00)		14 (53.80)		34 (43.60)		27 (40.30)		29 (48.33)		21 (36.80)		7 (58.33)		14 (70.00)		0.439	0.044
**RHC & LHC data**																						
RA (mmHg)	10.41	5.53	5.44	3.24	7.89	2.79	10.63	3.83	12.47	4.70	11.18	3.94	12.66	5.51	12.16	5.95	13.33	6.11	16.83	5.36	< 0.001	< 0.001
RV (mmHg)	9.26	6.55	4.43	3.87	7.16	3.62	9.09	5.74	11.19	6.44	9.81	6.25	12.05	6.86	11.84	6.67	9.58	6.57	13.89	6.35	< 0.001	< 0.001
sPAP (mmHg)	52.77	17.93	33.91	6.53	43.95	7.10	53.92	14.42	55.30	14.25	51.81	12.45	65.83	14.44	64.63	25.47	72.17	20.03	72.55	16.74	< 0.001	< 0.001
mPAP (mmHg)	33.64	11.77	19.95	3.42	27.40	2.66	34.92	8.76	35.79	8.13	33.27	6.76	42.10	89.76	41.07	9.34	50.50	11.61	49.70	8.49	< 0.001	< 0.001
dPAP (mmHg)	19.87	8.83	10.16	4.24	16.80	2.63	21.31	6.69	21.62	6.62	19.76	5.20	24.53	6.72	24.05	7.62	34.75	8.32	33.10	5.72	< 0.001	< 0.001
PCWP (mmHg)	21.12	9.16	11.09	4.03	13.05	1.70	20.42	5.47	25.14	7.19	22.39	6.61	26.92	7.05	25.91	8.79	25.33	7.62	31.65	6.44	< 0.001	< 0.001
DPG_PCWP_ (mmHg)	−1.23	5.69	−0.93	4.97	3.75	2.84	0.88	5.78	−3.53	4.43	−2.63	4.71	−2.38	5.27	−1.77	6.33	9.83	3.61	1.45	7.06	< 0.001	0.011
DPG_LVEDP_ (mmHg)	−1.76	10.65	−9.76	7.50	−2.95	7.77	9.46	7.25	−1.95	9.99	−5.73	8.08	3.03	8.74	0.28	7.81	14.08	9.99	13.10	6.63	< 0.001	< 0.001
TPG_PCWP_ (mmHg)	12.52	5.98	8.85	3.29	14.35	3.00	14.50	6.45	10.65	3.69	10.88	2.99	15.18	5.73	15.16	6.29	25.17	8.80	18.05	9.05	< 0.001	< 0.001
TPG_LVEDP_ (mmHg)	12.01	12.97	0.02	8.78	7.65	7.77	23.07	9.22	12.23	11.02	7.78	8.94	20.60	9.09	17.30	8.79	29.83	12.19	29.70	9.34	< 0.001	< 0.001
PVR (WU)	3.25	2.15	2.64	1.58	3.11	1.08	3.55	2.89	1.83	0.63	1.91	0.64	5.02	2.28	4.34	2.13	6.00	2.50	5.77	1.99	< 0.001	< 0.001
CO (L/min)	4.19	1.18	4.18	1.32	4.22	1.06	3.95	9.54	4.62	1.14	4.65	1.01	3.68	1.00	3.85	1.18	3.91	0.83	3.91	1.20	< 0.001	0.002
LVEDP	21.63	7.89	19.93	7.46	19.75	7.32	11.85	2.77	23.56	8.15	25.49	7.40	21.50	7.92	23.77	7.19	20.67	7.18	20.00	3.80	0.071	< 0.001
**Procedurale data**																						
Transfemoral approach—no. (%)	193 (85.80)		48 (87.30)		18 (90.00)		22 (84.60)		65 (83.30)		58 (86.60)		52 (86.66)		48 (84.20)		10 (83.33)		17 (85.00)		0.928	0.991
CoreValve—no. (%)	57 (25.30)		11 (20.00)		7 (35.00)		9 (34.60)		22 (28.20)		17 (25.40)		14 (23.33)		15 (26.30)		3 (25.00)		5 (25.00)		0.684	0.728
JenaValve—no. (%)	28 (12.40)		7 (12.70)		1 (5.00)		3 (11.50)		10 (12.80)		7 (10.40)		8 (13.33)		8 (14.00)		2 (16.66)		3 (15.00)		0.866	0.971
Edwards—no. (%)	138 (61.30)		37 (67.30)		12 (60.00)		14 (53.80)		45 (57.70)		43 (64.20)		37 (61.66)		32 (56.10)		7 (58.33)		12 (60.00)		0.858	0.676
Major vascular complications—no. (%)	25 (11.10)		8 (14.50)		2 (10.00)		2 (7.70)		6 (7.7)		4 (6.00)		7 (11.66)		8 (14.00)		2 (16.66)		3 (15.00)		0.736	0.469

**Table 2:** Tabular overview of regression analysis with regard to various, clinical characteristics. mPAP: mean pulmonary arterial pressure; BMI: body mass index; EF: ejection fraction.

	R	R^2^	F	B	B (SE)	Beta	*p*-Value
**Overall cohort**	0.210	0.044	10.292	0.244	0.076	0.210	0.002
**mPAP < 25 mmHg**	0.117	0.014	0.739	−0.063	0.074	−0.117	0.394
**mPAP ≥ 25 mmHg**	0.220	0.048	8.556	0.219	0.075	0.220	0.004
**Age < 80 years**	0.210	0.044	3.601	0.246	0.130	0.210	0.061
**Age ≥ 80 years**	0.195	0.038	5.678	0.226	0.095	0.195	0.018
**BMI < 30 kg/m^2^**	0.253	0.064	11.189	0.288	0.086	0.253	0.001
**BMI ≥ 30 kg/m^2^**	0.104	0.011	0.622	0.135	0.171	0.104	0.433
**Creatinine < 132 µmol/l**	0.238	0.057	9.320	0.266	0.087	0.238	0.003
**Creatinine ≥ 132 µmol/l**	0.357	0.127	7.012	0.540	0.204	0.357	0.011
**No Diabetes**	0.244	0.059	5.617	0.278	0.117	0.244	0.020
**Diabetes**	0.186	0.034	4.710	0.220	0.101	0.186	0.032
**No Hypertension**	0.090	0.008	0.148	0.151	0.392	0.090	0.705
**Hypertension**	0.224	0.050	10.697	0.251	0.077	0.224	0.001
**No Atrial Fibrillation**	0.207	0.043	6.613	0.233	0.090	0.207	0.011
**Atrial Fibrillation**	0.216	0.047	3.633	0.272	0.143	0.216	0.061
**Coronary Heart Disease < 2 vessels**	0.222	0.049	8.580	0.261	0.089	0.222	0.004
**Coronary Heart Disease ≥ 2 vessels**	0.163	0.027	1.532	0.182	0.147	0.163	0.221
**No COPD**	0.241	0.058	10.713	0.276	0.084	0.241	0.001
**COPD**	0.088	0.008	21.235	0.108	0.176	0.088	0.542
**No Myocardial Infarction**	0.221	0.049	9.923	0.252	0.080	0.221	0.002
**Myocardial Infarction**	0.095	0.009	0.248	0.126	0.252	0.095	0.622
**No Stroke**	0.202	0.041	8.119	0.237	0.083	0.202	0.005
**Stroke**	0.271	0.074	2.384	0.309	0.200	0.271	0.133
**NYHA < III**	0.027	0.001	0.019	0.036	0.265	0.027	0.893
**NYHA ≥ III**	0.267	0.071	14.126	0.308	0.082	0.267	< 0.001
**EF < 50%**	0.261	0.068	4.526	0.323	0.152	0.261	0.037
**EF ≥ 50%**	0.110	0.012	1.834	0.122	0.090	0.110	0.178
**Mitral Regurgitation < II°**	0.107	0.012	1.356	0.116	0.099	0.107	0.247
**Mitral Regurgitation ≥ II°**	0.326	0.106	11.514	0.397	0.117	0.326	0.001
**Tricuspid Regurgitation < II°**	0.284	0.081	9.664	0.337	0.108	0.284	0.002
**Tricuspid Regurgitation ≥ II°**	0.200	0.040	3.857	0.240	0.122	0.200	0.053

## Data Availability

The data presented in this study are available on request from the corresponding author.

## Abbreviations

ANOVA	Analysis of variances
AS	Aortic stenosis
borderlinepc-PH	Borderline post-capillary pulmonary hypertension
CO	Cardiac output
cpc-PH	Combined post-capillary pulmonary hypertension
dPAP	Diastolic pulmonary artery pressure
DPG	Diastolic pressure gradient
ESC	European Society of Cardiology
ipc-PH	Isolated post-capillary pulmonary hypertension
LHC	Left heart catheterization
LVEDP	Left ventricular end-diastolic pressure
LVEF	Left ventricular ejection fraction
mPAP	Mean pulmonary artery pressure
PCWP	Pulmonary capillary wedge pressure
PH	Pulmonary hypertension
prec-PH	Pre-capillary pulmonary hypertension
PVR	Pulmonary vascular resistance
RHC	Right hear catheterization
sPAP	Systolic pulmonary artery pressure
TAVI	Transcatheter aortic valve implantation
TPG	Transpulmonary pressure gradient
WHO	World Health Organization
